# Impact of Previous Stroke on Clinical Outcome in Elderly Patients With Nonvalvular Atrial Fibrillation: ANAFIE Registry

**DOI:** 10.1161/STROKEAHA.121.038285

**Published:** 2022-04-20

**Authors:** Takeshi Yoshimoto, Kazunori Toyoda, Masafumi Ihara, Hiroshi Inoue, Takeshi Yamashita, Shinya Suzuki, Masaharu Akao, Hirotsugu Atarashi, Takanori Ikeda, Ken Okumura, Yukihiro Koretsune, Wataru Shimizu, Hiroyuki Tsutsui, Atsushi Hirayama, Masahiro Yasaka, Hirofumi Maruyama, Satoshi Teramukai, Tetsuya Kimura, Yoshiyuki Morishima, Atsushi Takita, Takenori Yamaguchi

**Affiliations:** Department of Neurology (T. Yoshimoto, M.I.), National Cerebral and Cardiovascular Center, Suita, Japan.; Department of Cerebrovascular Medicine (K.T., T. Yamaguchi), National Cerebral and Cardiovascular Center, Suita, Japan.; Department of Clinical Neuroscience and Therapeutics, Hiroshima University, Japan (T. Yoshimoto).; Saiseikai Toyama Hospital, Toyama, Japan (H.I.).; The Cardiovascular Institute, Tokyo, Japan (T. Yamashita, S.S.).; Department of Cardiology, NHO Kyoto Medical Center, Kyoto, Japan (M.A.).; AOI Hachioji Hospital, Tokyo, Japan (H.A.).; Department of Cardiovascular Medicine, Toho University Faculty of Medicine, Tokyo, Japan (T.I.).; Division of Cardiology, Saiseikai Kumamoto Hospital Cardiovascular Center, Japan (K.O.).; NHO Osaka National Hospital, Japan (Y.K.).; Division of Cardiology, Nippon Medical School Department of Medicine, Tokyo, Japan (W.S.).; Department of Cardiovascular Medicine, Kyushu University Graduate School of Medical Science, Fukuoka, Japan (H.T.).; Osaka Police Hospital, Japan (A.H.).; Department of Cerebrovascular Medicine and Neurology, Cerebrovascular Center, NHO Kyushu Medical Center, Fukuoka, Japan (M.Y.).; Department of Biostatistics, Graduate School of Medical Science, Kyoto Prefectural University of Medicine, Japan (S.T.).; Primary Medical Science Department, Daiichi Sankyo, Tokyo, Japan (T.K., Y.M.).; Data Intelligence Department, Daiichi Sankyo Co., Ltd., Tokyo, Japan (A.T.).

**Keywords:** atrial fibrillation, cardioembolism, direct oral anticoagulants, elderly, intracranial hemorrhage, stroke

## Abstract

**Background:**

We determined the long-term event incidence among elderly patients with nonvalvular atrial fibrillation in terms of history of stroke/transient ischemic attack (TIA) and oral anticoagulation.

**Methods:**

Patients aged ≥75 years with documented nonvalvular atrial fibrillation enrolled in the prospective, multicenter, observational All Nippon Atrial Fibrillation in the Elderly Registry between October 2016 and January 2018 were divided into 2 groups according to history of stroke/TIA. The primary end point was the occurrence of stroke/systemic embolism within 2 years, and secondary end points were major bleeding and all-cause death within 2 years. Cox models were used to determine whether there was a difference in the hazard of each end point in patients with/without history of stroke/TIA, and in ischemic stroke/TIA survivors taking direct oral anticoagulants versus those taking warfarin.

**Results:**

Of 32 275 evaluable patients (13 793 women [42.7%]; median age, 81.0 years), 7304 (22.6%) had a history of stroke/TIA. The patients with previous stroke/TIA were more likely to be male and older and had higher hazard rates of stroke/systemic embolism (adjusted hazard ratio, 2.25 [95% CI, 1.97–2.58]), major bleeding (1.25, 1.05–1.49), and all-cause death (1.13, 1.02–1.24) than the other groups. Of 6446 patients with prior ischemic stroke/TIA, 4393 (68.2%) were taking direct oral anticoagulants and 1668 (25.9%) were taking warfarin at enrollment. The risk of stroke/systemic embolism was comparable between these 2 groups (adjusted hazard ratio, 0.90 [95% CI, 0.71–1.14]), while the risk of major bleeding (0.67, 0.48–0.94), intracranial hemorrhage (0.57, 0.39–0.85), and cardiovascular death (0.71, 0.51–0.99) was lower among those taking direct oral anticoagulants.

**Conclusions:**

Patients aged ≥75 years with nonvalvular atrial fibrillation and previous stroke/TIA more commonly had subsequent ischemic and hemorrhagic events than those without previous stroke/TIA. Among patients with previous ischemic stroke/TIA, the risk of hemorrhagic events was lower in patients taking direct oral anticoagulants compared with warfarin.

**REGISTRATION:**

URL: https://www.clinicaltrials.gov; Unique Identifier: UMIN000024006.

Atrial fibrillation (AF) is an independent risk factor for stroke, systemic embolism (SE) and all-cause death,^1,2^ and prior stroke/transient ischemic attack (TIA) increases the stroke risk of patients with AF by 2.5 times.^3^ Thus, preventing subsequent ischemic stroke (IS) and intracranial hemorrhage (ICH) in patients with AF with a history of stroke/TIA has become increasingly important. All the sub-analyses of 4 randomized controlled trials comparing direct oral anticoagulants (OACs) with warfarin in patients with nonvalvular AF (NVAF) reported higher incidences of stroke/SE and major bleeding in patients with previous stroke/TIA than in those without history of such events.^4–7^

The challenge in anticoagulant therapy in secondary prevention is the balance between the benefit of preventing ischemic events and the risk of bleeding, in particular, ICH. Both the risks of subsequent IS and ICH increase with increasing age.^8^ However, studies on the incidence of events and event risk factors in elderly patients with NVAF and previous stroke/TIA are limited.^9^ For example, patients with stroke aged ≥75 years comprised ≈40% of overall patients with stroke in the prior randomized controlled trials, and very elderly patients (≥85 years) represented a minority at 2% to 3%.^4–7^ To overcome these limitations, we designed a sub-analysis using data from the All Nippon AF in Elderly (ANAFIE) Registry, a nationwide, prospective registry study on elderly patients with NVAF.^10–13^ The aim was to compare the long-term event incidence between elderly patients with NVAF and previous stroke/TIA and those without a history of stroke. Additionally, the effectiveness of direct OACs (DOACs) was evaluated in patients with NVAF and previous stroke/TIA.

## Methods

The data supporting the present findings are available from the corresponding author on reasonable request.

### Study Population

The ANAFIE Registry (UMIN Clinical Trials Registry UMIN000024006) is a prospective, multicenter, observational study of elderly Japanese patients (aged ≥75 years) with NVAF. The rationale and full study design were published elsewhere.^10,14^ Briefly, all participants were registered from 1273 medical institutions throughout Japan between October 2016 and January 2018 and followed up for 2 years. Data were collected at baseline and at 1 and 2 years. Patients were censored when primary and secondary end points occurred. Ethics committee approvals were obtained from each institution, and all patients (or family members of patients with communication disorders, such as aphasia or cognitive impairment) provided written informed consent. The study was conducted in accordance with the Declaration of Helsinki. Data were reviewed by an independent data and safety monitoring committee. The present study conforms to the Strengthening the Reporting of Observational Studies in Epidemiology (STROBE) guidelines.^15^ A completed STROBE checklist is included in the Supplemental Material.

Patients who met the following criteria were included in the Registry: (1) aged ≥75 years at enrollment, (2) definitive diagnosis of NVAF established by electrocardiographic findings, and (3) ability to attend specified clinic visits. Exclusion criteria are described in Table S1. OACs use at enrollment was defined as either already taking an OAC at enrollment and continuing the OAC or starting an OAC at enrollment.

### End Point Events and Follow-Up Period

The primary end point of the present sub-analysis was the incidence of stroke/SE. The secondary end points were the incidence of the following events: major bleeding according to the International Society on Thrombosis and Hemostasis statement,^16^ IS, ICH, cardiovascular death, all-cause death, and net clinical outcome. Net clinical outcome was defined as a composite of stroke/SE, major bleeding, and all-cause death. The complete end points definitions have been published elsewhere.^10^ End point ascertainment was recorded by collecting at the patient’s most recent outpatient visit after the onset of the end point or, in some cases, by hospital visit records or reports from other medical institutions since the frequency of outpatient visits for registered patients varied depending on facility conditions and individual patients. As soon as the information about the end point was obtained, the investigators wrote it in the medical record and reported it to the research secretariat at the designated time 12 and 24 months later.

### Statistical Analysis

We summarized continuous variables as median (interquartile range), and categorical variables as frequencies and percentages. Baseline variables were compared among patients with and without previous stroke/TIA using the Wilcoxon rank-sum test or Pearson χ2 test, as appropriate. End points were assessed 2 years after obtaining informed consent. Primary and secondary end points were analyzed using the Kaplan-Meier method and described as rates per 100 person-years (PY) with 95% CIs. The Cox proportional hazards model was used to compare hazard ratios (HRs) by the history of stroke/TIA; HRs and 95% CIs were estimated using patients without previous stroke/TIA as a reference. We constructed the multivariable models of the primary and secondary end points. Since the number of each end point exceeded 400, as shown in the tables, it was statistically capable for us to include >20 variables for adjustment. Thus, all the following variables, which were significantly different between NVAF patients with and without previous stroke/TIA in a univariate regression model or known as established risk factors for the end points, were set as covariates: sex, age, body mass index, hypertension, diabetes, dyslipidemia, hyperuricemia, heart failure, severe hepatic disease, digestive diseases, active cancer, dementia, fall within 1 year, previous stroke subtype, history of major bleeding, history of myocardial infarction, history of thromboembolic disease, history of nonpharmacotherapy for NVAF, admission OACs, anti-platelet agents, proton pump inhibitor, or P-glycoprotein inhibitor at enrollment, polypharmacy and creatinine clearance.

Patients with previous IS/TIA or previous hemorrhagic stroke (HS) were compared with those without previous stroke/TIA. Patients having both previous IS and HS were considered as patients with previous IS/TIA.

Further, patients with previous IS/TIA were stratified into 3 groups (direct oral anticoagulant [DOAC], warfarin, and non-OAC [ie, those not receiving OACs]) according to OAC use at enrollment. The Cox proportional hazards model was used for intergroup comparison with the warfarin group as a reference.

Subgroup analyses in patients with previous IS/TIA were performed on the basis of patient characteristics, including sex, age (<85 and ≥85 years), current smoking, major bleeding, myocardial infarction, thromboembolism, type of AF (paroxysmal, persistent, and long-standing persistent/permanent), CHA_2_DS_2_-VASc score (2–3, 4–5, and 6–9 points), creatinine clearance (severe renal disease/dialysis/<30, ≥30–<50, and ≥50 mL/min). Cox models were used to test the interactions between treatments (DOAC versus warfarin) and to derive HRs for stroke/SE for patients with previous IS/TIA.

All statistical analyses were performed using SAS software, version 9.4 (SAS Institute, Inc, Cary, NC)

## Results

**Figure 1.**
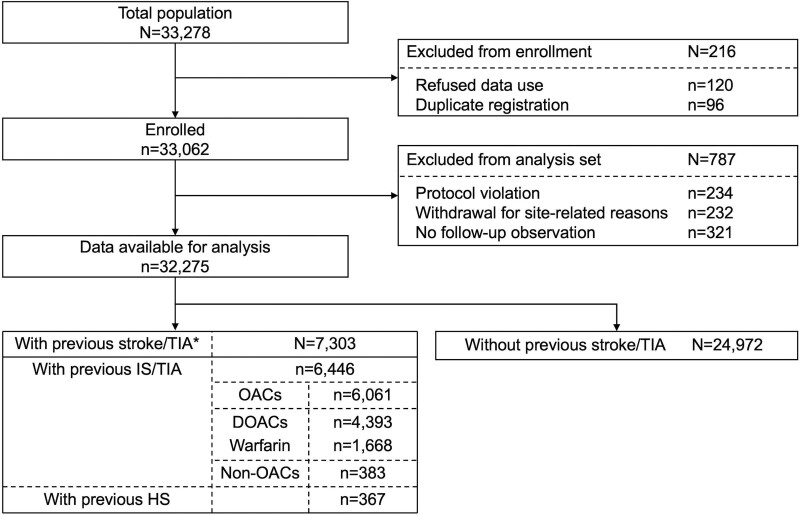
**Patient study flow.** *Includes patients of unknown stroke type (n=490). DOAC indicates direct oral anticoagulant; HS, hemorrhagic stroke; IS, ischemic stroke; OAC, oral anticoagulant; and TIA, transient ischemic attack.

Of the 33 062 patients enrolled, 787 were excluded due to protocol violation, withdrawal for site-related reasons, or loss to follow-up; the remaining 32 275 patients (13 793 women [42.7%]; median age, 81.0 years) were available for analysis. Of these, 7303 patients (22.6%) had a history of stroke/TIA (Figure 1). The baseline characteristics of the patients with and without previous stroke/TIA are summarized in Table 1. The patients with previous stroke/TIA were more likely to be male (62.8% versus 55.8%) and older (median 81.0 [78.0–85.0] versus 81.0 [77.0–85.0]), and had higher median CHADS_2_ (4 versus 2), CHA_2_DS_2_-VASc (6 versus 4), and HAS-BLED (3 versus 2) scores than those without previous stroke/TIA. At enrollment, 21 585 patients (66.9%) were taking DOACs and 8233 (25.5%) were taking warfarin. Patients with IS/TIA (n=6446) included 516 (7.1%) patients with TIA, and patients with the following stroke subtypes: large-artery atherosclerosis, 655 (11.0%); cardioembolism, 2377 (40.1%); small-vessel occlusion, 1436 (19.7%); and stroke of other determined/undetermined cause, 1765 (24.2%).

**Table 1.**
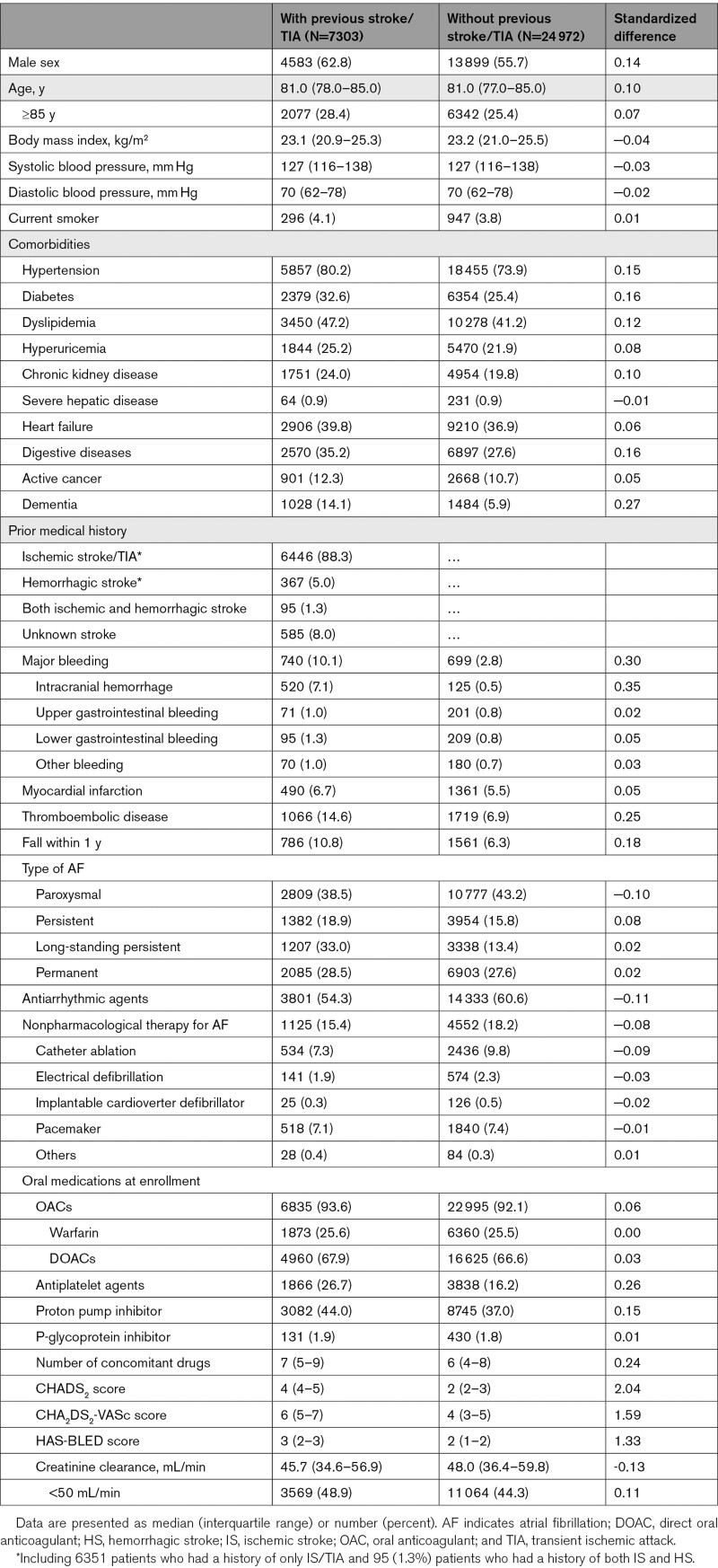
Baseline Demographic and Clinical Characteristics of Patients With and Without Previous Stroke or TIA

### Outcomes During the 2-Year Follow-Up

**Figure 2.**
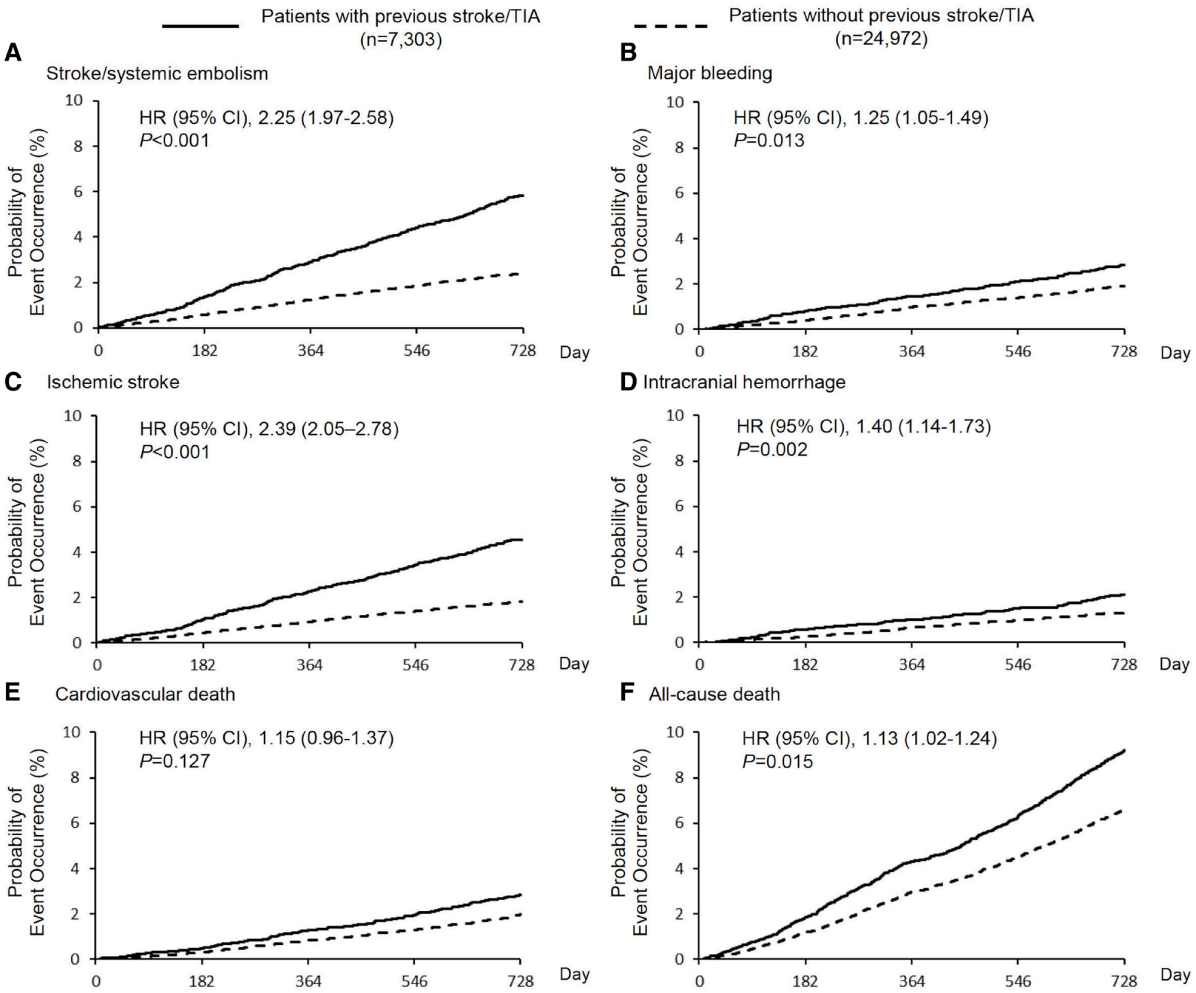
**Kaplan-Meier curves for primary and secondary end points in the total population.** Hazard ratios (HRs) of patients with previous stroke/transient ischemic attack (TIA) group are shown with reference to those without previous stroke/TIA. *P* values shown are for comparisons between groups with vs without previous stroke/TIA. HS indicates hemorrhagic stroke; and IS, ischemic stroke.

The mean follow-up period of patients with and without previous stroke/TIA was 1.86 and 1.89 years, respectively. Excluding those lost to follow-up, end points were assessed in 94.2% of patients in both groups. Compared with patients without previous stroke/TIA, patients with stroke/TIA had higher HRs of stroke/SE (primary efficacy outcome; 3.01/100 PY versus 1.23/100 PY; adjusted hazard ratio [HR] 2.25 [95% CI, 1.97–2.58]; Table 2, Figure 2A), major bleeding, IS, ICH, and all-cause death (Figure 2B through 2F). Thus, net clinical outcomes were higher in patients with previous stroke/TIA (7.84/100 PY versus 4.85/100 PY; adjusted HR, 1.35 [95% CI, 1.25–1.45]; Table 2) compared with those without previous stroke/TIA.

**Table 2.**
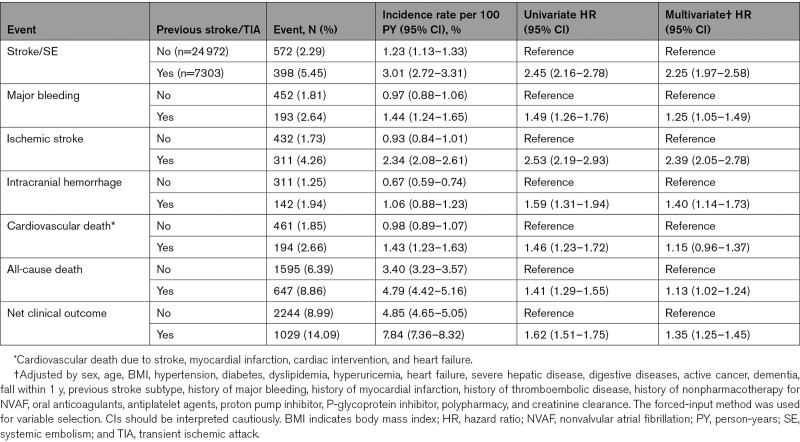
Incidence of Primary and Secondary End Points Between Patients With and Without Previous Stroke or TIA

### Outcomes by Type of Previous Stroke/TIA

Among patients with previous stroke/TIA, 87.0% (6351/7303) had a history of only IS/TIA, 3.7% (272/7303) had a history of only HS, 1.3% (95/7303) had a history of both IS and HS, and 8.0% (585/7303) had an unknown stroke type. The incidence of outcomes and the results of univariate and multivariate analyses in the Cox proportional hazard model by the type of previous stroke/TIA are shown in Figure 2 and Table S2. The risk of stroke/SE (adjusted HR, 2.33 [95% CI, 2.03–2.67]), major bleeding (HR, 1.26 [95% CI, 1.04–1.51]), IS (HR, 2.49 [95% CI, 2.13–2.91]), ICH (HR, 1.35 [95% CI, 1.08–1.68]), cardiovascular death (HR, 1.21 [95% CI, 1.01–1.45]), all-cause death (HR, 1.14 [95% CI, 1.03–1.26]), and net clinical outcome (HR, 1.37 [95% CI, 1.27–1.49]) were all higher in the patients with previous IS/TIA (n=6446) than in those without previous stroke/TIA (n=24 972). The risks of ICH (HR, 2.52 [95% CI, 1.20–5.26]) were higher in patients with previous HS (n=367) compared with those without previous stroke/TIA.

### Comparison of Anticoagulation Therapy in Patients With Previous IS/TIA

**Figure 3.**
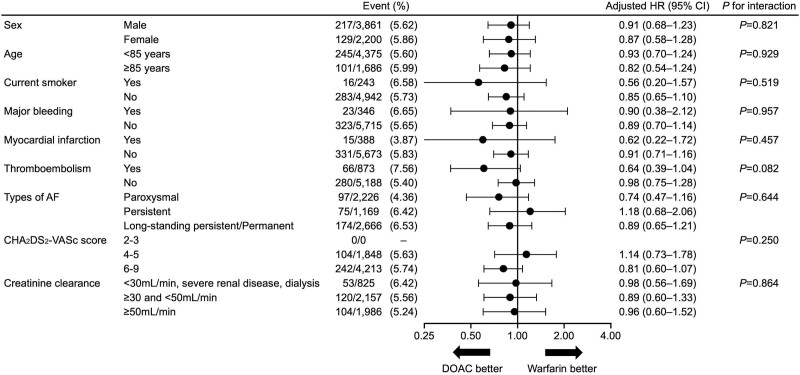
**Adjusted hazard ratios (HRs) for medication (direct oral anticoagulant [DOAC] vs warfarin) of stroke or systemic embolism in patients with previous ischemic stroke/transient ischemic attack.** AF indicates atrial fibrillation.

Of the 6446 patients with previous IS/TIA, 4393 (68.2%) were taking DOACs, and 1668 (25.9%) were taking warfarin at enrollment. Table 3 shows adjusted HRs of primary and secondary end points according to anticoagulant in patients with previous IS/TIA. The risk of major bleeding (adjusted HR, 0.67 [95% CI, 0.48–0.94]), ICH (HR, 0.57 [95% CI, 0.39–0.85]), cardiovascular death (HR, 0.71 [95% CI, 0.51–0.99]), and net clinical outcomes (HR, 0.85 [95% CI, 0.74–0.99]) were lower in the DOAC group than in the warfarin group. However, risk of stroke/SE, IS, and all-cause death was comparable between both groups. The subgroup analyses for the HRs of stroke/SE in patients with previous IS/TIA are shown in Figure 3. No significant interactions were observed between each patient characteristic and medication for stroke/SE.

**Table 3.**
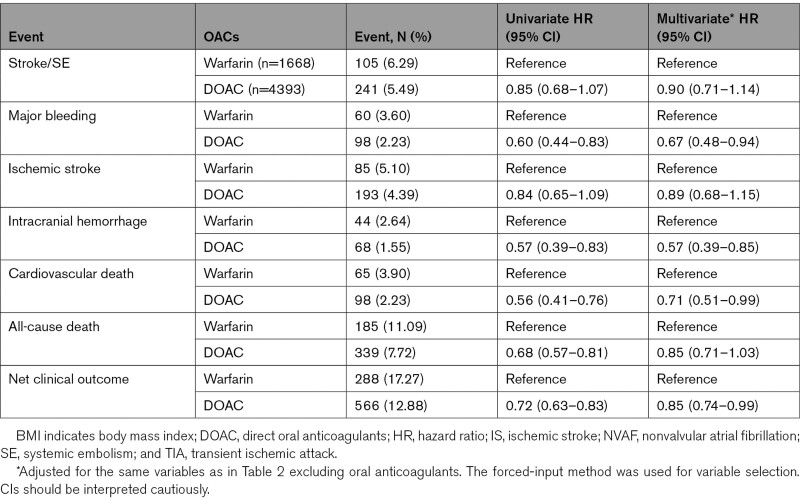
Incidence of Primary and Secondary End Points Between Patients With Previous IS/TIA Taking DOACs and Those Taking Warfarin

Incidences of primary and secondary end points of the patients in the non-OAC group are shown in Table S3; no significant differences relative to the warfarin group were observed.

## Discussion

The first major finding of this sub-analysis of ANAFIE Registry data was that elderly patients with previous stroke/TIA more commonly developed stroke/SE, major bleeding, IS, ICH, all-cause death, and net clinical outcome than those without previous stroke/TIA. The second was that in the patients with previous IS/TIA, the risk of major bleeding, ICH, and cardiovascular death was lower with DOAC treatment than with warfarin.

History of stroke/TIA more than doubled the risk of stroke/SE (HR 2.25) in the present cohort. Similar increases in the risk of stroke/SE were reported in patients with NVAF aged ≥75 years with a history of stroke/TIA from other Japanese registries. The HR was 2.27 in the J-ELD AF Registry,^17^ in which all participants used apixaban, and 2.42 in the nonanticoagulated participants of the Fushimi AF Registry.^18^ Both stroke history and aging are established risk factors of NVAF-associated stroke and components of the CHADS_2_ and CHA_2_DS_2_-VASc scores.^19,20^ Because elderly patients with NVAF are more likely to have a history of stroke than younger patients, secondary stroke prevention for older patients is critical.

Anticoagulation is the established and most effective therapy for secondary stroke prevention in patients with NVAF. However, both stroke history and aging are also established risk factors of anticoagulation-associated bleeding events and components of the HAS-BLED score.^21^ Thus, physicians should be careful when prescribing anticoagulation for elderly patients with NVAF and stroke history. There was a trend towards underuse of OACs in elderly patients with NVAF; the prevalence of OAC users was 48.4% in the Darlington AF Registry (mean age: 83.6 years) and 55.4% in Fushimi AF Registry (mean age: 81.8 years).^22,23^ The bleeding risk by OAC use for elderly patients would be overworried. In contrast, 93.6% of patients with stroke/TIA, whose median HAS-BLED score was 3, were treated with OACs in the present study. In our another registry for patients with NVAF and acute IS/TIA (SAMURAI-NVAF),^24^ 695/735 (94.7%) survivors aged ≥75 years were using OACs at the time of acute hospital discharge (unpublished data), indicating the widespread use of OACs for secondary prevention even among elderly patients. These high percentages seem to be desirable since anticoagulation is highly recommended for secondary stroke prevention in patients with AF. As the finding of relatively low bleeding risk with DOACs even in aged population has been widely reported, the percentage of OAC medication would increase. Another possible reason of the high percentage in ANAFIE was that participating physicians might have preferentially registered patients receiving anticoagulation who would likely benefit highly from stroke prevention.

An important finding of this subanalysis was the low incidence of bleeding complications among patients with a history of stroke (0.97/100 PY for major bleeding; 0.67/100 PY for ICH), despite a very high prevalence of OAC use and relatively high prevalence of anti-platelet use (26.7%). In previous studies on patients with NVAF aged ≥75 years, the annual incidence of major bleeding was generally >4%.^25–29^ A possible reason for the difference between this and other studies was that physicians participating in the ANAFIE Registry might have focused on controlling blood pressure and other factors relevant to preventing bleeding events. In this study, nearly three-quarters of patients receiving OACs were using DOACs. DOAC users showed lower incidences of bleeding events and deaths than warfarin users. The effectiveness in the present sub-analysis of DOACs in preventing bleeding complications in patients with stroke history compared with warfarin was consistent with the results of 4 major randomized controlled trials.^4–7^

The main strength of our study was that, to the best of our knowledge, it is the most extensive registration study of elderly NVAF patients undertaken to date, with a total of 32 275 patients including 7303 patients with previous stroke/TIA, and very low withdrawal rates during the 2-year follow-up period (2.3%). In addition, comprehensive data were available on stroke conditions before registration and end point events, which allowed the differentiation of IS from HS.

This study had several limitations. First, the present results might not be generalizable to elderly individuals with NVAF and a low OAC medication rate. Second, the interval from the previous stroke/TIA onset to registration was relatively variable. Intermixing patients registered early after stroke onset and during the chronic stage of stroke, and patients with a long history of OAC treatment before registration and those initiating OAC treatment at registration might complicate the interpretation of the results. Third, discontinuation of OAC or change to another OAC during the observation period was not assessed in this substudy. Finally, all the subjects were registered in Japan, the known country for longevity. Thus, the results might not be generalizable to other ethnicities.

## Conclusions

Elderly Japanese patients with NVAF and previous stroke/TIA had higher HRs of stroke/SE, major bleeding, and all-cause death than those without previous stroke/TIA. Among patients with previous IS/TIA, the risk of hemorrhagic events was lower among patients treated with DOACs compared with warfarin.

## Article Information

### Acknowledgments

We thank IQVIA Services Japan K.K. and EP-CRSU for their partial support in the conduct of this Registry, and Keyra Martinez Dunn, MD, of Edanz (www.edanz.com) for providing medical writing support. In addition, the authors thank Daisuke Chiba, of Daiichi Sankyo (DS) Co, Ltd, for supporting the preparation of the article.

### Sources of Funding

DS Co, Ltd, supported the ANAFIE Registry. The sponsor was involved in the study design, planning of the data analysis, data interpretation, and decision to submit the article for publication, but was not directly involved in data management, direct access, or statistical analysis. The corresponding author had full access to all data and was responsible for the submission for publication.

### Disclosures

All the following conflicts are outside the submitted work. Yoshimoto reports lecturer’s fees from Nippon Boehringer Ingelheim (NBI) and Takeda. Dr Toyoda reports lecturer’s fees from Daiichi-Sankyo (DS), Bayer, Takeda, and Bristol-Myers Squibb (BMS). Dr Ihara reports lecturer’s fees from DS and Eisai, and grant support from Panasonic, GE Precision Healthcare LLC, BMS, and Shimadzu Corporation. Dr Inoue reports remuneration from DS, Bayer, and BMS. Dr Yamashita reports research funding from DS, BMS, and Bayer, article fees from DS, and BMS, and remuneration from DS, Bayer, Pfizer Japan, BMS, and Ono Pharmaceutical. Dr Suzuki reports research funding from DS, and remuneration from DS and BMS. Dr Akao reports research funding from DS and Bayer, and remuneration from BMS, NBI, Bayer, and DS. Dr Atarashi reports remuneration from DS. Dr Ikeda reports research grants from DS, Medtronic Japan, and Japan Lifeline and honoraria from Ono Pharma, Bayer, DS, BMS, and Pfizer, and was a member of the advisory board for Bayer, BMS, and DS. Dr Okumura reports remuneration from NBI, DS, Johnson & Johnson, and Medtronic. Dr Koretsune reports remuneration from DS, Bayer, and NBI. Dr Shimizu reports research funding from DS, BMS, and NBI, and patent royalties/licensing fees from DS, Pfizer Japan, BMS, Bayer, and NBI. Dr Tsutsui reports research funding from DS, Mitsubishi Tanabe Pharma, NBI, and IQVIA Services Japan, remuneration from DS, Bayer, NBI, Pfizer Japan, Otsuka Pharmaceutical, and Mitsubishi Tanabe Pharma, scholarship funding from DS, Mitsubishi Tanabe Pharma, and Teijin Pharma, and consultancy fee from Novartis Pharma, Pfizer Japan, Bayer, NBI, and Ono Pharmaceutical. Hirayama reports participated in a course endowed by Boston Scientific Japan, and has received research funding from DS and Bayer, and remuneration from Bayer, DS, BMS, NBI, Sanofi, Astellas Pharma, Sumitomo Dainippon Pharma, Amgen Astellas BioPharma, and AstraZeneca, and patent royalties/licensing fees from Toa Eiyo. Dr Yasaka reports research funding from NBI, and remuneration from NBI, DS, Bayer, BMS, Pfizer Japan, and CSL Behring. Dr Teramukai reports research funding from NBI and remuneration from DS, Sanofi, Takeda, Chugai Pharmaceutical, Solasia Pharma, Bayer, Sysmex, Nipro, NapaJen Pharma, Gunze, and Atworking. T. Kimura has stock and is an employee of DS. Dr Morishima and A. Takita are employees of DS. Dr Maruyama reports speaker fees from Eisai, Pfizer, DS, BMS, NBI, and Bayer, and research support from Eisai and DS. Dr Yamaguchi reports acted as an advisory board member of DS and received remuneration from DS, and BMS.

### Supplemental Material

Checklist

Tables S1–S3

## Supplementary Material



## References

[1] Kannel WB Wolf PA Benjamin EJ Levy D. Prevalence, incidence, prognosis, and predisposing conditions for atrial fibrillation: population-based estimates. Am J Cardiol. 1998;82(8A):2N–9N. doi: 10.1016/s0002-9149(98)00583-9 10.1016/s0002-9149(98)00583-9 9809895

[2] Schnabel RB Yin X Gona P Larson MG Beiser AS McManus DD Newton-Cheh C Lubitz SA Magnani JW Ellinor PT. 50 year trends in atrial fibrillation prevalence, incidence, risk factors, and mortality in the Framingham Heart Study: a cohort study. Lancet. 2015;386:154–162. doi: 10.1016/S0140-6736(14)61774-8 25960110 10.1016/S0140-6736(14)61774-8 PMC4553037

[3] McGrath ER Kapral MK Fang J Eikelboom JW Conghaile A O’Conghaile A Canavan M O’Donnell MJ; Investigators of the Ontario Stroke Registry. Association of atrial fibrillation with mortality and disability after ischemic stroke. Neurology. 2013;81:825–832. doi: 10.1212/WNL.0b013e3182a2cc15 23902702 10.1212/WNL.0b013e3182a2cc15

[4] Diener HC Connolly SJ Ezekowitz MD Wallentin L Reilly PA Yang S Xavier D Di Pasquale G Yusuf S; RE-LY Study Group. Dabigatran compared with warfarin in patients with atrial fibrillation and previous transient ischaemic attack or stroke: a subgroup analysis of the RE-LY trial. Lancet Neurol. 2010;9:1157–1163. doi: 10.1016/S1474-4422(10)70274-X 21059484 10.1016/S1474-4422(10)70274-X

[5] Hankey GJ Patel MR Stevens SR Becker RC Breithardt G Carolei A Diener HC Donnan GA Halperin JL Mahaffey KW; ROCKET AF Steering Committee Investigators. Rivaroxaban compared with warfarin in patients with atrial fibrillation and previous stroke or transient ischaemic attack: a subgroup analysis of ROCKET AF. Lancet Neurol. 2012;11:315–322. doi: 10.1016/S1474-4422(12)70042-X 22402056 10.1016/S1474-4422(12)70042-X

[6] Easton JD Lopes RD Bahit MC Wojdyla DM Granger CB Wallentin L Alings M Goto S Lewis BS Rosenqvist M; ARISTOTLE Committees and Investigators. Apixaban compared with warfarin in patients with atrial fibrillation and previous stroke or transient ischaemic attack: a subgroup analysis of the ARISTOTLE trial. Lancet Neurol. 2012;11:503–511. doi: 10.1016/S1474-4422(12)70092-3 22572202 10.1016/S1474-4422(12)70092-3

[7] Rost NS Giugliano RP Ruff CT Murphy SA Crompton AE Norden AD Silverman S Singhal AB Nicolau JC SomaRaju B; ENGAGE AF-TIMI 48 Investigators. Outcomes with edoxaban versus warfarin in patients with previous cerebrovascular events: findings from ENGAGE AF-TIMI 48 (effective anticoagulation with factor Xa next generation in atrial fibrillation-thrombolysis in myocardial infarction 48). Stroke. 2016;47:2075–2082. doi: 10.1161/STROKEAHA.116.013540 27387994 10.1161/STROKEAHA.116.013540

[8] Wolf PA Abbott RD Kannel WB. Atrial fibrillation: a major contributor to stroke in the elderly. the Framingham study. Arch Intern Med. 1987;147:1561–1564. doi: 10.1001/archinte.1987.00370090041008 3632164

[9] Okumura K Yamashita T Suzuki S Akao M; J-ELD AF Investigators. A multicenter prospective cohort study to investigate the effectiveness and safety of apixaban in Japanese elderly atrial fibrillation patients (J-ELD AF Registry). Clin Cardiol. 2020;43:251–259. doi: 10.1002/clc.23294 31737921 10.1002/clc.23294 PMC7068106

[10] Inoue H Yamashita T Akao M Atarashi H Ikeda T Okumura K Koretsune Y Shimizu W Tsutsui H Toyoda K. Prospective observational study in elderly patients with non-valvular atrial fibrillation: rationale and design of the All Nippon AF In the Elderly (ANAFIE) Registry. J Cardiol. 2018;72:300–306. doi: 10.1016/j.jjcc.2018.02.018 29625717 10.1016/j.jjcc.2018.02.018

[11] Kanemaru K Yoshimoto T Inoue H Yamashita T Akao M Atarashi H Ikeda T Okumura K Koretsune Y Shimizu W; All Nippon Atrial Fibrillation in the Elderly (ANAFIE) Group. Baseline characteristics of elderly Japanese patients aged ≥75 years with non-valvular atrial fibrillation and a history of stroke- ANAFIE registry. Circ J. 2020;84:516–523. doi: 10.1253/circj.CJ-19-0974 31983727 10.1253/circj.CJ-19-0974

[12] Koretsune Y Yamashita T Akao M Atarashi H Ikeda T Okumura K Shimizu W Tsutsui H Toyoda K Hirayama A. Baseline demographics and clinical characteristics in the all nippon AF in the elderly (ANAFIE) registry. Circ J. 2019;83:1538–1545. doi: 10.1253/circj.CJ-19-0094 31168044 10.1253/circj.CJ-19-0094

[13] Yamashita T Suzuki S Inoue H Akao M Atarashi H Ikeda T Okumura K Koretsune Y Shimizu W Tsutsui H. Two-year outcomes of more than 30 000 elderly patients with atrial fibrillation: results from the All Nippon AF In the Elderly (ANAFIE) registry. Eur Heart J Qual Care Clin Outcomes. 2021;8:202–213. doi: 10.1093/ehjqcco/qcab025 10.1093/ehjqcco/qcab025 PMC8888123 33822030

[14] Yasaka M Yamashita T Akao M Atarashi H Ikeda T Koretsune Y Okumura K Shimizu W Tsutsui H Toyoda K. Background characteristics and anticoagulant usage patterns of elderly non-valvular atrial fibrillation patients in the ANAFIE registry: a prospective, multicentre, observational cohort study in Japan. BMJ Open. 2021;11:e044501. doi: 10.1136/bmjopen-2020-044501 10.1136/bmjopen-2020-044501 PMC7942257 34006033

[15] von Elm E Altman DG Egger M Pocock SJ Gøtzsche PC Vandenbroucke JP; STROBE Initiative. The Strengthening the Reporting of Observational Studies in Epidemiology (STROBE) statement: guidelines for reporting observational studies. Ann Intern Med. 2007;147:573–577. doi: 10.7326/0003-4819-147-8-200710160-00010 17938396 10.7326/0003-4819-147-8-200710160-00010

[16] Schulman S Kearon C; Subcommittee on Control of Anticoagulation of the Scientific and Standardization Committee of the International Society on Thrombosis and Haemostasis. Definition of major bleeding in clinical investigations of antihemostatic medicinal products in non-surgical patients. J Thromb Haemost. 2005;3:692–694. doi: 10.1111/j.1538-7836.2005.01204.x 15842354 10.1111/j.1538-7836.2005.01204.x

[17] Okada M Inoue K Tanaka N Sakata Y Akao M Yamashita T Suzuki S Okumura K; J-ELD AF Investigators. Clinical outcomes of very elderly patients with atrial fibrillation receiving on-label doses of Apixaban: J-ELD AF registry subanalysis. J Am Heart Assoc. 2021;10:e021224. doi: 10.1161/JAHA.121.021224 34323123 10.1161/JAHA.121.021224 PMC8475673

[18] Yamashita Y Hamatani Y Esato M Chun YH Tsuji H Wada H Hasegawa K Abe M Lip GYH Akao M. Clinical characteristics and outcomes in extreme elderly (age ≥ 85 years) japanese patients with atrial fibrillation: the Fushimi AF registry. Chest. 2016;149:401–412. doi: 10.1378/chest.15-1095 26181726 10.1378/chest.15-1095

[19] Gage BF Waterman AD Shannon W Boechler M Rich MW Radford MJ. Validation of clinical classification schemes for predicting stroke: results from the National Registry of Atrial Fibrillation. JAMA. 2001;285:2864–2870. doi: 10.1001/jama.285.22.2864 11401607 10.1001/jama.285.22.2864

[20] Lip GY Nieuwlaat R Pisters R Lane DA Crijns HJ. Refining clinical risk stratification for predicting stroke and thromboembolism in atrial fibrillation using a novel risk factor-based approach: the euro heart survey on atrial fibrillation. Chest. 2010;137:263–272. doi: 10.1378/chest.09-1584 19762550 10.1378/chest.09-1584

[21] Pisters R Lane DA Nieuwlaat R de Vos CB Crijns HJ Lip GY. A novel user-friendly score (HAS-BLED) to assess 1-year risk of major bleeding in patients with atrial fibrillation: the Euro Heart Survey. Chest. 2010;138:1093–1100. doi: 10.1378/chest.10-0134 20299623 10.1378/chest.10-0134

[22] Ogawa H Senoo K An Y Shantsila A Shantsila E Lane DA Wolff A Akao M Lip GYH. Clinical features and prognosis in patients with atrial fibrillation and prior stroke: comparing the Fushimi and Darlington AF registries. EBioMedicine. 2017;18:199–203. doi: 10.1016/j.ebiom.2017.03.022 28330631 10.1016/j.ebiom.2017.03.022 PMC5405156

[23] Mazurek M Shantsila E Lane DA Wolff A Proietti M Lip GYH. Secondary versus primary stroke prevention in atrial fibrillation: insights from the Darlington atrial fibrillation registry. Stroke. 2017;48:2198–2205. doi: 10.1161/STROKEAHA.116.016146 28679859 10.1161/STROKEAHA.116.016146

[24] Toyoda K Arihiro S Todo K Yamagami H Kimura K Furui E Terasaki T Shiokawa Y Kamiyama K Takizawa S; SAMURAI Study Investigators. Trends in oral anticoagulant choice for acute stroke patients with nonvalvular atrial fibrillation in Japan: the SAMURAI-NVAF study. Int J Stroke. 2015;10:836–842. doi: 10.1111/ijs.12452 25581108 10.1111/ijs.12452 PMC4964913

[25] Patti G Lucerna M Pecen L Siller-Matula JM Cavallari I Kirchhof P De Caterina R. Thromboembolic risk, bleeding outcomes and effect of different antithrombotic strategies in very elderly patients with atrial fibrillation: a sub-analysis from the PREFER in AF (PREvention oF Thromboembolic Events-European Registry in Atrial Fibrillation). J Am Heart Assoc. 2017;6:e005657. doi: 10.1161/JAHA.117.005657 28736385 10.1161/JAHA.117.005657 PMC5586290

[26] Senoo K An Y Ogawa H Lane DA Wolff A Shantsila E Akao M Lip GY. Stroke and death in elderly patients with atrial fibrillation in Japan compared with the United Kingdom. Heart. 2016;102:1878–1882. doi: 10.1136/heartjnl-2016-309741 27312001 10.1136/heartjnl-2016-309741

[27] Halperin JL Hankey GJ Wojdyla DM Piccini JP Lokhnygina Y Patel MR Breithardt G Singer DE Becker RC Hacke W; ROCKET AF Steering Committee and Investigators. Efficacy and safety of rivaroxaban compared with warfarin among elderly patients with nonvalvular atrial fibrillation in the Rivaroxaban Once Daily, Oral, Direct Factor Xa Inhibition Compared With Vitamin K Antagonism for Prevention of Stroke and Embolism Trial in Atrial Fibrillation (ROCKET AF). Circulation. 2014;130:138–146. doi: 10.1161/CIRCULATIONAHA.113.005008 24895454 10.1161/CIRCULATIONAHA.113.005008

[28] Halvorsen S Atar D Yang H De Caterina R Erol C Garcia D Granger CB Hanna M Held C Husted S. Efficacy and safety of apixaban compared with warfarin according to age for stroke prevention in atrial fibrillation: observations from the ARISTOTLE trial. Eur Heart J. 2014;35:1864–1872. doi: 10.1093/eurheartj/ehu046 24561548 10.1093/eurheartj/ehu046 PMC4104493

[29] Kato ET Giugliano RP Ruff CT Koretsune Y Yamashita T Kiss RG Nordio F Murphy SA Kimura T Jin J. Efficacy and safety of edoxaban in elderly patients with atrial fibrillation in the ENGAGE AF-TIMI 48 trial. J Am Heart Assoc. 2016;5:e003432. doi: 10.1161/JAHA.116.003432 27207971 10.1161/JAHA.116.003432 PMC4889207

