# Supplementary material for: Impact of Previous Stroke on Clinical Outcome in Elderly Patients With Nonvalvular Atrial Fibrillation: ANAFIE Registry
Source: Stroke. 2022 Apr 20;53(8):2549–58. doi: 10.1161/STROKEAHA.121.038285 (PMC9311295; doi:10.1161/STROKEAHA.121.038285)
Supplement: Supplementary file 1 [file str-53-2549-s001.pdf]

Supplemental Material

Impact of previous stroke on clinical outcome in elderly patients with non-valvular atrial fibrillation: ANAFIE Registry

Table S1. Exclusion criteria (page 2)

Table S2. Univariate and multivariate analysis (Cox proportional hazard model) according to history of stroke/TIA (pages 3–4)

Table S3. Incidence of primary and secondary endpoints in patients with previous IS/TIA in the warfarin and non-OAC groups (page 5)

Table S1. Exclusion criteria

1)	Definitive diagnosis of cardiac valvular disease such as mitral stenosis
2)	Artificial heart valve replacement, involving either mechanical or tissue valve prostheses
3)	Recent cardiovascular events within 1 month prior to enrollment. including stroke, myocardial infarction, cardiac intervention, heart failure requiring hospitalization, or any bleeding leading to hospitalization
4)	Life expectancy of less than 1 year
5)	Participation deemed inappropriate by the investigator at each institution

Table S2. Univariate and multivariate analysis (Cox proportional hazard model) according to history of stroke/TIA

Event	History of stroke/TIA	Event, n (%)	Univariate HR (95% CI)	*P*-value	Multivariate† HR (95% CI)	*P*-value
Stroke/SE	None (n=24,972)	572 (2.29)	reference		reference	
	IS/TIA (n=6,446)	362 (5.62)	2.53 (2.22–2.88)	<0.001	2.33 (2.03–2.67)	<0.001
	HS (n=367)	14 (5.15)	2.33 (1.37–3.97)	0.002	1.54 (0.85–2.80)	0.153
Major bleeding	None	452 (1.81)	reference		reference	
	IS/TIA	167 (2.59)	1.46 (1.22–1.74)	<0.0001	1.26 (1.04–.51)	0.015
	HS	12 (4.41)	2.52 (1.42–4.47)	0.002	1.28 (0.67–2.44)	0.451
Ischemic stroke	None	432 (1.73)	reference		reference	
	IS/TIA	287 (4.45)	2.65 (2.28–3.07)	<0.001	2.49 (2.13–2.91)	<0.001
	HS	5 (1.84)	1.09 (0.45–2.62)	0.853	0.73 (0.29–1.86)	0.510
Intracranial hemorrhage	None	311 (1.25)	reference		reference	
	IS/TIA	119 (1.85)	1.51 (1.22–1.87)	<0.001	1.35 (1.08–1.68)	0.008
	HS	11 (4.04)	3.38 (1.85–6.16)	<0.001	2.52 (1.20–5.26)	0.014
Cardiovascular death*	None	461 (1.85)	reference		reference	
	IS/TIA	180 (2.79)	1.54 (1.30–1.83)	<0.001	1.21 (1.01–1.45)	0.038
	HS	3 (1.10)	0.61 (0.20–1.91)	0.397	0.33 (0.10–1.06)	0.062
All-cause death	None	1,595 (6.39)	reference		reference	
	IS/TIA	576 (8.94)	1.43 (1.30–1.57)	<0.001	1.14 (1.03–1.26)	0.009
	HS	26 (9.56)	1.53 (1.04–2.26)	0.030	1.00 (0.65–1.52)	0.984
Net clinical outcome	None	2,244 (8.99)	reference		reference	
	IS/TIA	919 (14.26)	1.64 (1.52–1.77)	<0.001	1.37 (1.27–1.49)	<0.001
	HS	39 (14.34)	1.66 (1.21–2.28)	0.002	1.04 (0.73–1.47)	0.833

* Cardiovascular death due to stroke, myocardial infarction, cardiac intervention, and heart failure.

† Adjusted for sex, age, BMI, hypertension, diabetes mellitus, dyslipidemia, hyperuricemia, heart failure, severe hepatic disease, digestive diseases, active cancer, dementia, fall within 1-year, previous stroke subtype, history of major bleeding, history of myocardial infarction, history of thromboembolic disease, history of non-pharmacotherapy for NVAF, oral anticoagulants, anti-platelet agents, proton pump inhibitor, P-glycoprotein inhibitor, polypharmacy, and creatinine clearance. The forced-input method was used for variable selection.

BMI indicates body mass index; CI, confidence interval; HR, hazard ratios; HS, hemorrhagic stroke; IS, ischemic stroke; NVAF, non-valvular atrial fibrillation; SE, systemic embolism; TIA, transient ischemic attack.

Table S3. Incidence of primary and secondary endpoints in patients with previous IS/TIA in the warfarin and non-OAC groups

Event	OACs	Event, n (%)	Univariate HR (95% CI)	*P*-value	Multivariate† HR (95% CI)	*P*-value
Stroke/SE	Warfarin	111 (5.93)	reference		reference	
	Non-OAC	22 (4.70)	0.81 (0.51–1.28)	0.368	0.94 (0.59–1.52)	0.809
Major bleeding	Warfarin	66 (3.52)	reference		reference	
	Non-OAC	11 (2.35)	0.68 (0.36–1.29)	0.853	0.73 (0.37–1.41)	0.342
Ischemic stroke	Warfarin	88 (4.70)	reference		reference	
	Non-OAC	15 (3.21)	0.70 (0.40–1.20)	0.193	0.84 (0.48–1.49)	0.557
Intracranial hemorrhage	Warfarin	50 (2.67)	reference		reference	
	Non-OAC	9 (1.92)	0.74 (0.36–1.50)	0.397	0.67 (0.32–1.40)	0.288
Cardiovascular death*	Warfarin	68 (3.63)	reference		reference	
	Non-OAC	21 (4.49)	1.27 (0.78–2.07)	0.340	1.11 (0.65–1.87)	0.708
All-cause death	Warfarin	196 (10.46)	reference		reference	
	Non-OAC	65 (13.89)	1.36 (1.03–1.80)	0.031	1.30 (0.96–1.75)	0.086
Net clinical outcome	Warfarin	307 (16.39)	reference		reference	
	Non-OAC	81 (17.31)	1.07 (0.84–1.37)	0.580	1.14 (0.88–1.47)	0.321

* Cardiovascular death due to stroke, myocardial infarction, cardiac intervention, and heart failure.

† Adjusted for the same variables as in Supplemental Table II. The forced-input method was used for variable selection.

BMI indicates body mass index; CI, confidence interval; HR, hazard ratios; IS, ischemic stroke; NVAF, non-valvular atrial fibrillation; OAC, oral anticoagulants; SE, systemic embolism; TIA, transient ischemic attack.
